# A Galectina-3 (Biomarcador de Fibrose Miocárdica) Prediz a Progressão na Doença de Chagas?

**DOI:** 10.36660/abc.20201162

**Published:** 2021-02-19

**Authors:** Roberto Coury Pedrosa

**Affiliations:** 1 Hospital Universitário Clementino Fraga FilhoInstituto do Coração Edson SaadUniversidade Federal do Rio de JaneiroRio de JaneiroRJBrasilDepartamento de Cardiologia, Hospital Universitário Clementino Fraga Filho / Instituto do Coração Edson Saad – Universidade Federal do Rio de Janeiro, Rio de Janeiro, RJ - Brasil

**Keywords:** Galectina 3, Apoptose, Fibrose Endomiocárdica, Doença de Chagas, Biomarcadores

Nas últimas décadas, a epidemiologia da Doença de Chagas (DCh) mudou significativamente, como consequência da urbanização e da migração.^1^ No Brasil, até o momento, é a doença negligenciada com a maior carga, da qual ~30% dos indivíduos evoluirá para a estágio sintomático de ruptura do tecido em 20 a 30 anos após a infecção^2^ (cerca de 2% ao ano evoluem da forma indeterminada da doença para a forma cardíaca, de acordo com um estudo atual).^3^

A inflamação miocárdica persistente e a fibrose representam as principais características patológicas da DCh que estariam relacionadas com a sua progressão.^4,5^ O uso de biomarcadores de fibrose miocárdica para prever a progressão em pacientes com função normal ou quase normal do VE é certamente importante na DCh, onde fatores de risco tradicionais, como a FEVE, podem não ser tão úteis. No entanto, estudos têm demonstrado o valor dos biomarcadores de fibrose miocárdica na progressão da patogênese da DCh.^6^

Nesta edição, Fernandes et al.,^7^ apresentam dados que contribuem para o nosso entendimento relacionado à carga da fibrose miocárdica em diferentes estágios da DCh. Para tanto, os autores avaliaram a presença (ou não) da Galectina-3 (Gal-3), um biomarcador de fibrose miocárdica, em diferentes estágios da doença comparados à um grupo controle e se ela está associada a mortalidade ou necessidade de transplante de coração no estágio mais avançado da doença. Para esse propósito, foram realizados 2 estudos com desenhos distintos. Inicialmente, a fim de estratificar os grupos pelo status de cardiomiopatia chagásica (CC) utilizando um desenho de estudo transversal, 330 pacientes soropositivos para *T. cruzi *(187 sem cardiomiopatia; 46 com CC/ECG anormal e FEVE >50%; e 97 com CC/ECG anormal com FEVE <50%) foram incluídos, além de 153 controles soronegativos (pareados por idade e sexo). Desses pacientes soropositivos, 97 com formas cardíacas mais graves de CC fizeram parte do estudo longitudinal prospectivo censurado até o evento (mortalidade ou necessidade de transplante cardíaco).

Os resultados foram resumidos e analisados nos diferentes grupos. A mediana de idade foi de 49 ± 9,2 anos, com mediana de seguimento de 58 meses. Pacientes com doença de Chagas sem cardiomiopatia (n = 187) e aqueles com cardiomiopatia e FEVE >50% (n = 46) tinham níveis de Gal-3 semelhantes aos de controles saudáveis (n = 153), mas aqueles com cardiomiopatia e FEVE <50% (n = 97) apresentaram níveis de Gal-3 significativamente maiores do que os controles saudáveis (p = 0,0001). Uma correlação significativa foi observada entre os níveis de Gal-3 e FEVE (r_s_ = -0,16, p = 0,001). Foram observados eventos em 28 pacientes (29%). Em pacientes com cardiopatia e FEVE <50%, o modelo de regressão linear ajustado mostrou uma associação significativa entre os níveis de Gal-3 e morte ou transplante cardíaco durante um seguimento de cinco anos (*Hazard ratio* - HR 3,11; IC 95% = 1,21– 8,04; p = 0,019).

Os autores concluíram que, em pacientes com a forma cardíaca, níveis mais elevados de Gal-3 estavam significativamente associados a formas graves da doença e a uma maior taxa de mortalidade em longo prazo, o que significa que podem ser utilizados de forma eficaz para identificar pacientes de alto risco.

Entretanto, os principais achados de Fernandes et al.,^7^ foram os seguintes: primeiro, os níveis de Gal-3 não mostraram nenhuma diferença entre indivíduos normais e pacientes com DCh sem cardiomiopatia e com cardiomiopatia / FEVE >50%. Esta observação sugere que os níveis de Gal-3 nesta amostra de pacientes não puderam ser utilizados como um marcador de progressão da doença miocárdica. O segundo achado importante deste estudo foi que os níveis mais elevados de Gal-3 estavam significativamente associados às formas graves da doença e a uma maior taxa de mortalidade em longo prazo. Esse resultado não é exclusivo da DCh, pois achados semelhantes foram observados nos estudos de Nagase et al.^8^ e Spinale.^9^

A interpretação desses resultados deve considerar o pequeno número de pacientes com seguimento de cinco anos e experiência em centro único já citados pelos autores. No grupo de pacientes com cardiomiopatia e FEVE <50%, possivelmente foram utilizados diferentes graus de comprometimento miocárdico, já que havia um amplo espectro de FEVE (variando de 20% a 40%) levando a diferentes perfis de pacientes incluídos nos grupos.^7^ É evidente que a taxa de eventos foi baixa e significativamente inferior à da coorte de alto risco de Rassi (taxa de mortalidade anual de 5,8% vs. 12,6%); assim, os resultados das análises multivariadas, que incluíram cinco variáveis independentes, devem ser considerados com cautela devido à provável supersaturação do modelo.

Um dos maiores desafios da DCh é a dificuldade da identificação, em um estágio inicial, os indivíduos infectados que farão parte dos 20% a 30% que podem desenvolver cardiomiopatia. Infelizmente, no momento, não há como prever essa possível progressão. Assim, encontrar biomarcadores confiáveis de progressão da doença significaria o maior salto na história da DCh desde sua descoberta em 1909 pelo Dr. Carlos Chagas. Por esse motivo, existem inúmeros grupos de pesquisa que se dedicam à busca de biomarcadores derivados do hospedeiro e do *T. cruzi* .^10^ Isso significaria um avanço no manejo da DCh.
