# Supplementary material for: A Galectina-3 (Biomarcador de Fibrose Miocárdica) Prediz a Progressão na Doença de Chagas?
Source: Arq Bras Cardiol. 2021 Feb 19;116(2):257–8. [Article in Portuguese] doi: 10.36660/abc.20201162 (PMC7909985; doi:10.36660/abc.20201162)
Supplement: Translation

# Translation: Does Galectin-3 (Myocardial Fibrosis Biomarker) Predict Progression in Chagas Disease?

**Authors:** Roberto Coury Pedrosa

**Affiliations:** 1 BrazilDepartamento de Cardiologia, Hospital Universitário Clementino Fraga Filho / Instituto do Coração Edson Saad – Universidade Federal do Rio de Janeiro, Rio de Janeiro, RJ - Brazil

**Keywords:** Galectin 3, Apoptosis, Endomyocardial Fibrosis, Chagas Disease Biomarkers

In recent decades, the epidemiology of Chagas disease (Chd) has changed significantly as a consequence of urbanization and migration.^1^ In Brazil, to date, it is the neglected disease with the highest burden, of which ~30% of individuals will progress to the symptomatic tissue disruptive stage within 20 – 30 years after being infected^2^ (around 2% per year progress from the indeterminate to the cardiac form, according to a contemporary study).^3^

Persistent myocardial inflammation and fibrosis represent the main pathological characteristics of Chd that would be correlated with its progression.^4,5^ The use of myocardial fibrosis biomarkers to predict progression in patients with normal or near normal LV function is certainly important in Chd, where traditional risk factors, such as LVEF, may not be as useful. Nevertheless, studies have shown the value of myocardial fibrosis biomarkers in the progression of Chd pathogenesis.^6^

In this edition, Fernandes et al.^7^ present data that contributes to our understanding related to the burden of myocardial fibrosis in different stages of Chd. To this end, the authors assessed the presence (or not) of Galectin-3 (Gal-3), a myocardial fibrosis biomarker, in different stages of the disease compared to a control group and whether it is associated with mortality or need for a heart transplant in the most advanced stage of the disease. For this purpose, 2 studies with different designs were carried out. Initially, in order to stratify the groups by the Chagas cardiomyopathy (CC) status using a cross-sectional study design, 330 patients seropositive for *T. cruzi* (187 without cardiomyopathy; 46 CC-abnormal ECG and LVEF>50%; and 97 CC-abnormal ECG with LVEF<50%) were included, in addition to 153 seronegative controls (matched by age and gender). Of these seropositive patients, 97 with more severe cardiac forms of CC were part of the prospective longitudinal study censored until the event (mortality or need for a heart transplant).

The results were summarized and analysed in the different groups. The median age was 49 ± 9.2 years, with a median follow-up of 58 months. Chagas disease patients without cardiomyopathy (n = 187) and those with cardiomyopathy and LVEF >50% (n = 46) had Gal-3 levels similar to those of healthy controls (n = 153), but those with cardiomyopathy and LVEF <50% (n = 97) had significantly higher Gal-3 levels than the healthy controls (p = 0.0001). A significant correlation was observed between Gal-3 levels and LVEF (r_s_=-0.16, p=0.001). Events were observed in 28 patients (29%). In patients with cardiopathy and LVEF< 50%, the adjusted linear regression model showed a significantly association between Gal-3 levels and death or heart transplantation during a five-year follow-up (Hazard ratio - HR 3.11; 95% CI = 1.21– 8.04; p = 0.019). The authors concluded that in patients with the cardiac form, higher Gal-3 levels were significantly associated with severe forms of the disease and a higher long-term mortality rate, which means that they can be effectively used to identify high-risk patients.

Nevertheless, the major findings of Fernandes et al.^7^ were as follows: First, Gal-3 levels did not show any difference between normal individuals and Chd patients without cardiomyopathy and with cardiomyopathy / LVEF >50%. This observation suggests that Gal-3 levels in this patient sample could be not used as a marker of myocardial disease progression . The second important finding of this study was that the higher Gal-3 levels were significantly associated with the severe forms of the disease and a higher long-term mortality rate. This result is not unique to Chd, as similar findings were seen in the studies by Nagase et al.^8^ and Spinale.^9^

The interpretation of these results should consider the small number of patients who had a five-year follow-up and single center experience already quoted by the authors. In the group of patients with cardiomyopathy and LVEF <50%, different degrees of myocardial involvement were possibly used, since there was a wide spectrum of LVEF (ranging from 20% to 40%) leading to different patient profiles included in the groups.^7^ It is noticeable that the rate of events was low and significantly inferior to that of the Rassi high risk cohort (annual mortality rate of 5.8% vs. 12.6%); thus, the results from the multivariate analyses, which included 5 independent variables, should be taken with caution due to likely model oversaturation.

One of the mayor challenges in Chd is the difficulty in identifying at an early stage the infected subjects who will be part of those 20% to 30% who might develop cardiomyopathy. Unfortunately, at present there is no way to predict this possible progression. Thus, finding reliable biomarkers of disease progression would mean the greatest leap forward in the history of Chd since its discovery in 1909 by Dr. Carlos Chagas. For this reason, there are numerous research groups devoted to the search of both host-derived and T. cruzi-derived biomarkers.^10^ This would mean a breakthrough in the management of Chd
